# A Lidar Point Cloud Based Procedure for Vertical Canopy Structure Analysis And 3D Single Tree Modelling in Forest

**DOI:** 10.3390/s8063938

**Published:** 2008-06-12

**Authors:** Yunsheng Wang, Holger Weinacker, Barbara Koch

**Affiliations:** Dept. of Remote Sensing and Landscape Information Systems, University of Freiburg, Germany; E-mails: yunsheng.wang@felis.uni-freiburg.deholger.weinacker@felis.uni-freiburg.debarbara.koch@felis.uni-freiburg.de

**Keywords:** Lidar, Point Cloud, Single Tree, Crown, 3D Modelling

## Abstract

A procedure for both vertical canopy structure analysis and 3D single tree modelling based on Lidar point cloud is presented in this paper. The whole area of research is segmented into small study cells by a raster net. For each cell, a normalized point cloud whose point heights represent the absolute heights of the ground objects is generated from the original Lidar raw point cloud. The main tree canopy layers and the height ranges of the layers are detected according to a statistical analysis of the height distribution probability of the normalized raw points. For the 3D modelling of individual trees, individual trees are detected and delineated not only from the top canopy layer but also from the sub canopy layer. The normalized points are resampled into a local voxel space. A series of horizontal 2D projection images at the different height levels are then generated respect to the voxel space. Tree crown regions are detected from the projection images. Individual trees are then extracted by means of a pre-order forest traversal process through all the tree crown regions at the different height levels. Finally, 3D tree crown models of the extracted individual trees are reconstructed. With further analyses on the 3D models of individual tree crowns, important parameters such as crown height range, crown volume and crown contours at the different height levels can be derived.

## 1. Introduction

The vertical canopy structures of stands are of high interest in forest management. It is also of high interest for assessment of the regeneration success and biodiversity aspects. The knowledge of the vertical canopy structure improves regression models for estimation of wood volume and biomass and the information of sub canopy layer will benefit the management of new growth in forest. Lidar is especially suitable to reproduce the vertical canopy structure of forest stands due to its capability of three-dimensional measurements with high accuracy. The utilization of Lidar in forest investigation can be generally divided into canopy height distribution based and single tree detection based. Several approaches for Canopy height distribution detection have been achieved in the past few years (Naesset, 2002; Lim et al, 2003; Holmgren and Jonsson, 2004), these researches are mostly focused on the forest characteristics of the top canopy layer. Concerning the vertical distribution of canopy layers, researches based on large-footprint Lidar data with continuous waveform have been accomplished by Lefsky et al.(1999) and Harding et al.(2001). Andersen et al.(2003) have presented a method for estimating vertical canopy structure of forest through a group of regression functions based on field investigations. As for single tree delineation, the majority of the existed algorithms are Digital Surface Model (DSM) based (Hyyppä and Inkinen 1999; Persson et al. 2002; Koch et al. 2006). Trees are delineated according to the features of crowns on the DSM, thus the individual trees in the lower canopy layer whose crowns are covered by the top canopy layer cannot be detected. Besides the detection of individual trees, Pyysalo and Hyyppä (2002) have provided a process for reconstructing tree crowns, with a previous knowledge of the location and the crown size of a single tree, raw points belonging to the tree are extracted, the height of the tree, the height of the crown and the average radius of the crown at different heights are derived. Furthermore, a new full-waveform based algorithm for detecting tree stems has been delivered by Reitberger et al. (2007).

In this paper, we will present a procedure for both vertical canopy structure analysis and single tree modelling in forest based on Lidar raw point clouds. The major task of vertical canopy structure analysis is to detect the number of main canopy layers and the height range of each canopy layer. A more detailed study of the spatial features of canopies is performed by the single tree modelling process. Individual trees are detected not only from the upper canopy layer but also from the sub canopy layer in between the ground and upper canopy layer. Shapes of individual tree crowns are then delineated and 3D models of tree crowns are reconstructed.

The test area of this study is in “Kuernacher Wald”, southern Germany. The size of the area is 2.7km*2.8km, where 97.5% is covered by forest. The dominant tree species in the region are: Spruce-65.3%; Beech-25.9%; Sycamore-3.5%; Fir-2% and others 3.9%. The average stand volume is 390 m^3^/ha and stand age is from 5 to over 100 years. The Lidar data is a first-last echo data with point density of 4∼7 points/m^2^, which is delivered by Toposys.

## 2. Vertical canopy structure Analysis

### 2.1 Normalized Point Cloud and Grids

**Figure 1.**
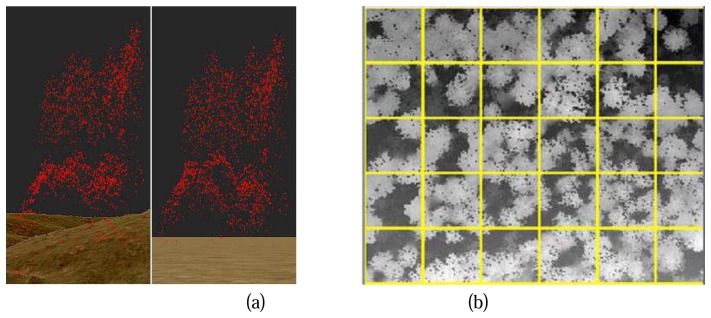
(a) Comparison between original Lidar raw point cloud and normalized point cloud; Left: original Lidar raw point cloud and the DTM; Right: normalized point cloud over a zero height level surface; (b) Grids over DSM (part of Kuernacher Wald).

To get the absolute object height from the raw points, the influence of terrain must be eliminated (Figure 1.(a) Left). A raster Digital Terrain Model (DTM) is used for the normalization of raw point heights. The DTM is generated from the raw point clouds based on the active contour algorithm implemented by TreesVis, a software for LIDAR data processing developed by Department of Remote Sensing and Landscape Information Systems (FeLis) (Weinacker et al., 2004).

As shown in Figure 1.(a) Left, raw points are projected above the DTM, the height difference between a raw point and its correspondent terrain is marked as the normalized height of the point. A normalized point cloud is then generated (Figure 1. (a) Right), where point heights of the normalized point cloud represent the absolute heights of the objects.

Due to the huge amount of data, it is not possible to analyze all the normalized points for the whole study area in one step. Therefore the whole area of research is segmented by a gird (Figure 1. (b)). Each cell of the grid is then a study cell, the size of the cell can be flexible, an area between 400m^2^ (20m*20m) to 40000 m^2^ (200m*200m) is seemingly found good for the calculation according to our experiences. 20m*20m is the chosen size in our study case according to the size of inventory sample plots in Germany (radius = 12m). Further analyses are carried out for each study cell separately. The results of each study cell will be assembled for the whole study area at the end.

### 2.2 Detection of Canopy Layers

#### 2.2.1 Height Distribution Probability of Normalized Points

With a statistical process of the normalized points, a height distribution probability function *ф(h)* can be derived. According to the physical feature of Lidar data, most of the reflected points are located in canopy layers in the forest area. Therefore there should be an obvious increase of reflected points at each canopy layer. Thus, the problem of canopy layer detection can be transferred to a salient curve detection based on the height distribution probability function *ф(h)*.

**Figure 2.**
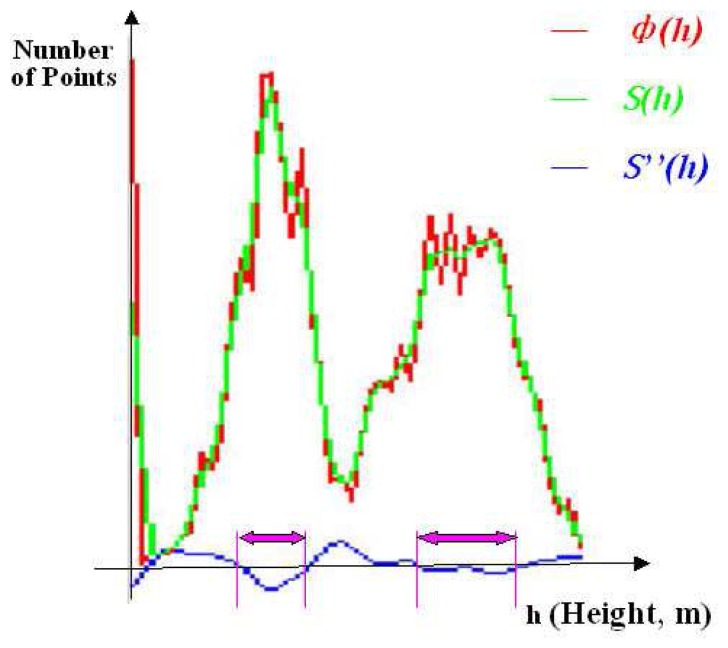
Relationship between *ф(h)*, *S(h)* and *S″(h)*, the ranges of canopy layers are extracted at the height intervals where *S″(h)* < 0.

As presented in figure 2,. to reduce the influence of slight amplitude movements on the function, *ф(h)* is firstly smoothed with a gaussian function, a smoothed function *S(h)* is generated, the second derivative *S″ (h)* is then calculated for the smoothed function *S(h)*.

The magnitude of the second derivative is a useful criterion to detect salient curves. With each *S″ (h)* =0, there is an inflexion point of function *S(h)* at *h*. At the intervals of *h* where *S″ (h)*<0, there must be salient curves of function *S(h)*, the intervals of *h* are considered as height ranges of canopy layers.

#### 2.2.2 Attributes of Canopy Layers

**Figure 3.**
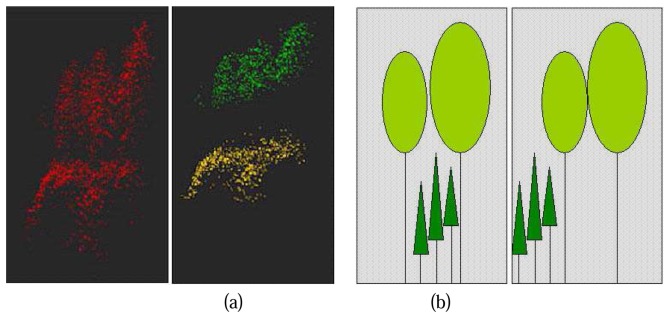
(a) Points within the ranges of the detected canopies with comparison of original point cloud; Left, Normalized point cloud; Right, Points within detected canopy ranges; (b) Two different forest stands with same height distribution probability density function; Left: Double layered forest stand; Right: Single layer forest stand with trees of mixed height

The number of canopy layers in each study cell and the height range of each canopy layer are the main attributes derived from the vertical canopy structure analysis. The range of a canopy layer starts from the height where the most rapid increase of point amounts occurred, the end of the range is marked at the height where the sharpest decrease of point amounts takes place (Figure 3.(a)).

As illustrated in figure 3.(b), although there is no difference in height distribution of normalized points between the two cases, the spatial relationship of canopy layers is distinct. In the left case, the canopy layers are overlapped, such kind of situation can be considered as a real double layered forest stand. On the contrary, the canopy layers in the right case are separated, this is actually a stand of trees with mixed heights. To detect the real duple layer stand, the basic concept is to check the horizontal distribution of the canopy layers.

**Figure 4.**
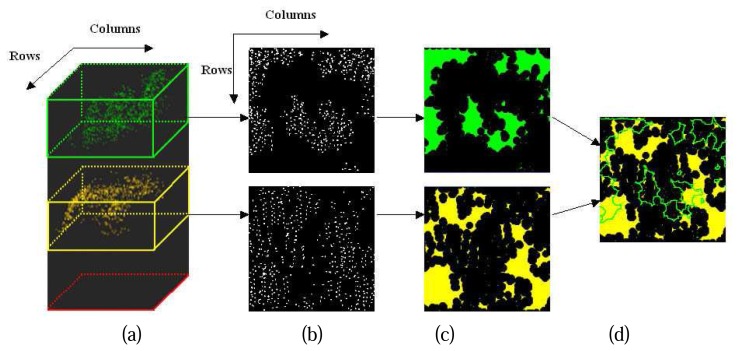
Inspection of horizontal distribution of canopy layers, (a) 3D spatial distribution of normalized points at different canopy layers; (b) 2D horizontal projections of normalized points at different canopy layers; (c) Horizontal distributions of different canopy layers; (d) examination of overlapping area between two canopy layers

As shown in Figure 4.(a) and (b), suppose there is a screen horizontally suspended over the study cell, project all the points of a canopy layer onto the screen, the 3D spatial feature of the canopy layer will be reduced to 2D on the screen. The 2D horizontal distributions of the canopy layers are then extracted associated with their 2D spatial features on the screen resulting from a morphological analysis (Figure 4.(c)). In a real duple layer stand, the overlapping area between two canopy layers should be larger than half area of upper canopy layer or half area of sub canopy layer (Figure 4.(d)). Further details about the 2D horizontal projections will be presented in the third chapter.

**Figure 5.**
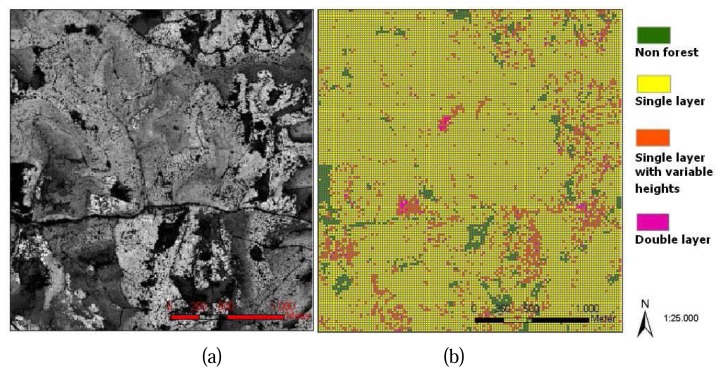
(a) DSM of the study area (Kuernacher Wald); (b) Results of vertical canopy structure analysis (ArcGIS *.shp file).

With an implementation of the process for all study cells, the distribution of canopy layers in the whole study area can be mapped, a 2D GIS shape file is generated (figure 5. (b)). Study cells whose canopy heights are lower than 2m are considered to be non-forest cells. Other information such as number of canopy layers and height range of each canopy layer is stored in the attributes table. Figure 5. (a) shows the DSM of the study area, compare to the results of vertical canopy layer analysis (figure 5. (b)), several stand features on the DSM are recognizable from the results of vertical canopy structure analysis such as the non forest areas, the homogenous areas and the mixed stand areas.

## 3. 3D Single tree Modelling

### 3.1 2D Horizontal Projection Images

The inspiration of 3D single tree modelling comes from the inspection of horizontal distribution of canopies. If we can project the canopy layers in 2D in order to investigate the horizontal distribution of the canopies, it is possible to get the 2D horizontal distribution of individual tree crowns at the different height levels when more 2D horizontal projections are available.

A local voxel space is defined for each study cell to describe the 3D spatial features of the normalized points, all the normalized points within the study cell are remapped into the voxel space.

**Figure 6.**
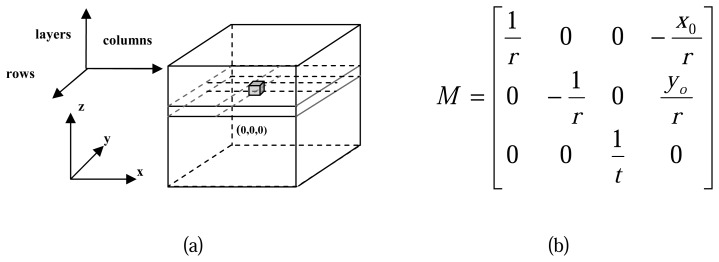
(a) Local voxel space with real world coordinate system (x, y, z) and voxel coordinate system (rows, columns, layers); (b) Definition of transformation matrix M.

For the transformation of normalized points from real world coordinate system (x, y, z) to the voxel coordinate system (rows, columns, layers), a transformation matrix ***M*** is defined as shown in figure 6.(b), of which: *r* represents the raster resolution of the horizontal surface in the voxel space; *t* represents the thickness of each layer in the voxel space; *x_o_y_o_* are the coordinates of the local origin, namely the *x, y* coordinate in real world coordinate system of the upper-left corner in the study cell; For each normalized point in real world *P(x,y,z)*, there is a correspondent point in voxel space *P′(row, column, layer)*, the relationship between *P* and *P′* can be defined with function:[*row column layer*]*^T^* = *Round* (*M*× [*x y z* 1]*^T^*)

According to the density of raw point cloud and the scale of *r* and *t* in transformation matrix ***M***, it is possible that several normalized points are located inside a same voxel.

**Figure 7.**
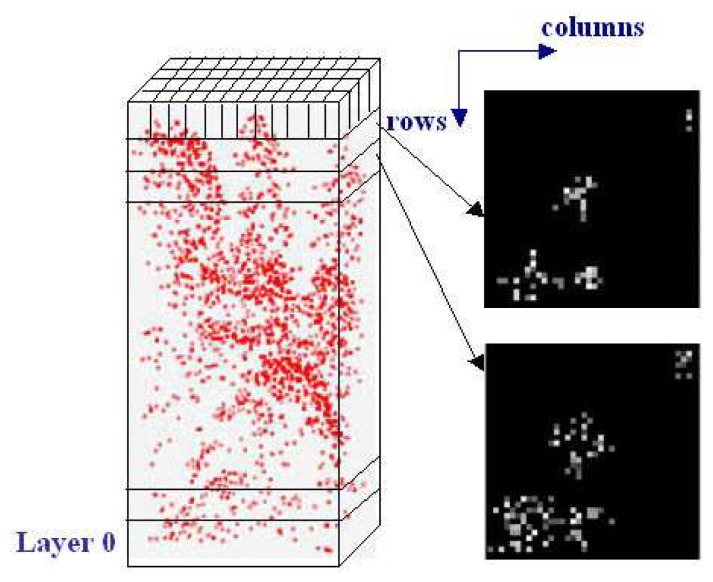
Normalized points in local voxel space and two examples of projection images of two vertical neighbouring layers of the voxel space.

To simplify the computational complexity, a series of 2D horizontal projection images are introduced into the further analyses as an instrument to record the voxel space. Taking all the voxels of a single layer out of the voxel space, the voxels of the layer can be regarded as pixels of an image. The number of normalized points within each voxel is marked as a gray value of the correspondent pixel in the image. A 2D horizontal projection image of the selected layer is then generated (Figure 7.).

Points of each individual tree crown will present a cluster feature on the horizontal projection image. The presence of the cluster features is highly related to the horizontal resolution *r* and the thickness *t* of the voxel space. The values of parameter *r* and *t* in transformation matrix ***M*** rely on the density of the raw point cloud. According to our experiments, a horizontal resolution of 0.5 meter and a thickness of 1 meter are the ideal values for dataset with 5∼12 points per m^2^.

### 3.2 Modelling of Single Tree Crowns

The clusters on the horizontal projection image at each layer represent the distribution of tree crowns in the correspondent height level. Therefore an individual tree crown should be visible at the same location of several vertical neighboring layers. The basic concept of single tree extraction is to trace the presence of crown outlines on the projection images from top to bottom through projection images at the different height levels. Outlines of the crown at the upper layers will present a cluster feature on the projection image, thus the task of detecting individual tree turns to be extracting cluster feature of treetop then tracing the crown outline from top to bottom.

#### 3.2.1 Delineation of individual tree crown contours

**Figure 8.**
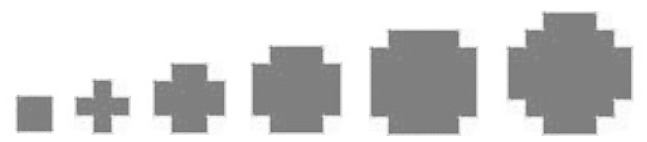
Structuring elements used in hierarchical morphological process.

Potential tree crown contours in each layer are delineated based on the cluster features on the correspondent projection image. A hierarchical morphological opening and closing process with a group of predefined structuring elements (Figure 8.) is performed.

It can be assumed that the amount of raw points should be higher where the crowns present, especially at the crown outlines. Considering the projection image, a higher gray value of a pixel represents a higher point density in its correspondent voxel. Thus a higher ranking significance should be assigned to the pixel with higher gray value and a larger neighborhood of the pixel should be kept. The morphological process begins with the brightest pixels on the projection image, these pixels are taken as seeds and closed by the largest structuring element, then opened by the smallest structuring element, potential tree crown regions are then extracted based on the brightest pixels. Similar process is fulfilled with pixels of other gray value levels, the lower gray value the pixels have, the smaller structuring element is used for closing and the bigger structuring element is used for opening. Finally, potential regions from different gray value levels at same neighborhoods are merged. Levels of gray value are defined according to the histogram of the projection image at non-zero gray value area, of which highest level: *α*>=80%; middle level: 20% <*α*<80%; lowest level: *α*<= 20%.

**Figure 9.**
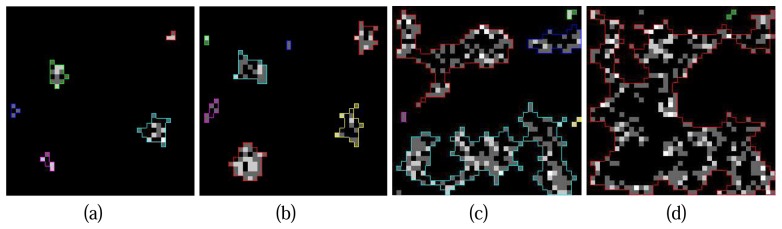
A real case of hierarchical morphological based crown contour extraction at different height level, (a) crown contours at top layer (39m); (b) crown contours at 38m; (c) crown contours at 36m; (d) crown contours at 35m.

Figure 9 illustrates a series of crown contours at the different height levels delineated by the hierarchical morphological algorithm. It is obvious that the contours of individual crowns are easy to delineate at the beginning (Figure 9. (a) and (b)) because of the concentration of Lidar points at tree tops. Situations turn to be challenging when the process goes to lower layers since crowns are not solid with Lidar points and neighbouring crowns conjunct, individual crowns are hardly to detect in these cases (Figure 9. (c)). The situation can be even worse at the height level where all the neighbouring tree crowns touch each other (Figure 9.(d)), the clusters on the projection image might be considered as a whole thus the delineated contour is useless. To solve this problem an improvement of the hierarchical morphological algorithm is necessary.

#### 3.2.2 Improved tree crown contour delineation

The process of this stage is inspired by the DSM based single tree delineation algorithms that are mostly based on morphological pouring or watershed whose most significant section is to stop the region growing when the neighboring regions touch each other.

**Figure 10.**
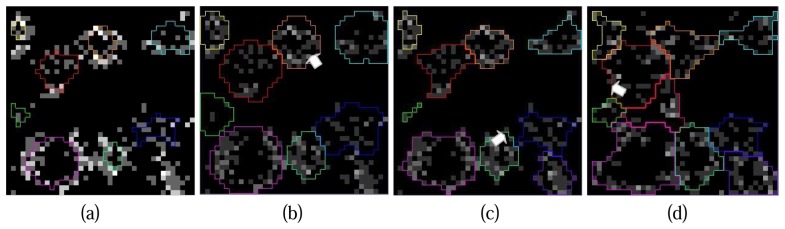
Simulation of pouring, (a) Crown contours from upper layer as seed areas (contours at 37m height level is overlapped on the projection image at 36m height level in this case); (b) Region growing of seed areas at current layer (36m), split when neighbour regions overlap; (c) Crown contours (36m) with the expanded seed regions as reference; (d) Crown contours delineated at 35m with contours from 36m as reference.

A Simulation of pouring is included in the tree crown contour delineation process. As being presented in figure 10. (a), crown contours from the higher height level is now taken as a reference. The crown contours from the upper layer is expended according to the cluster features on the projection image, the enlargement is stopped when the neighboring regions conjunct. The white arrow in figure 10.(b) points the position of the conjunctions.

The hierarchical morphological algorithm is parallel implemented inside and outside the enlarged reference regions. Comparing figure 10. (c) and (d) to figure 9 and (d), the benefits from the reference regions are distinct. The utilization of reference regions will not influence the emergence of new treetops at sub height level due to the parallel process in and outside the reference regions. White arrows in figure 10.(c) and (d) indicate the appearance of new tree tops.

#### 3.2.3 Pre-order forest traversal

In computer science, forest traversal or more generally tree traversal refers to the process of visiting each node in a forest or tree data structure systematically. In our case, tree crown regions on the layers at the different height levels can be considered to be nodes at different levels of a forest data structure, crown regions on the top layer are the prime root nodes of the forest. A pre-order forest traversal process is fulfilled to visit all the crown regions of the forest.

**Figure 11.**
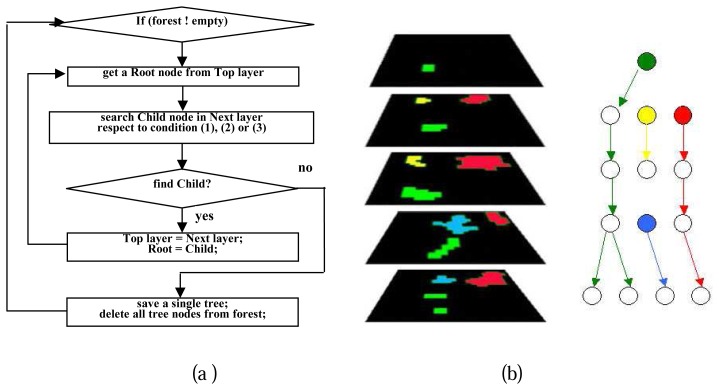
(a) Main process of single tree extraction; (b) Demonstration of single tree extraction during a pre-order forest traversal process; Left: tree crown regions in different layers; Right: Result of single tree extraction, each tree is marked with a different colour, the root node of each tree is filled up with the same colour as the corresponding tree.

Individual tree crowns are extracted during the forest traversal process by grouping the vertical neighboring crown contours from layers at the different height levels. The main procedures of single tree extraction is illustrated in figure 11.(a), a real case of single tree extraction is demonstrated in figure 11.(b). For each root node, namely the top region of each crown, the conditions of the existence of a child node, namely a vertical neighboring crown region in next layer, are listed as follows:

(1) Ai/Ar>Ca

(2) Ai/Ac>Ca

(3) D<Min(Rr,Rc)

where

Ai = intersection area of root node and child node
Ar = area of root node
Ac = area of child node
Ca = constant criteria in interval [0.5, 1.0], for which 0.8 is an ideal value in our study case
D = distance between centre points of root node and child node
Rr = average radius of root node
Rc = the average radius of the child node

It is not necessary to satisfy all the three conditions, two regions are recognized to be neighboring regions when any one of the conditions is met.

#### 3.2.4 3D models of tree crowns

**Figure 12.**
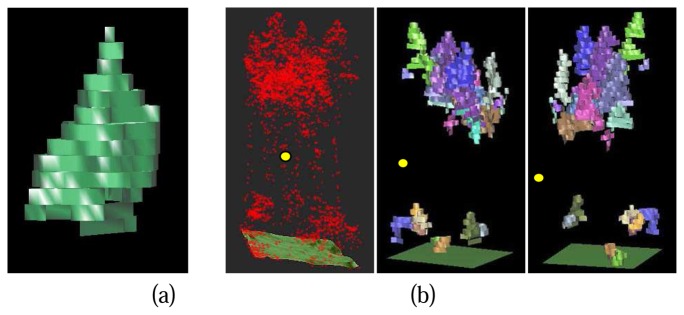
(a) Virtual Reality Modelling Language (VRML) prismatic model of an individual crown; (b)VRML prismatic model of individual tree crowns in a single study cell comparison with raw points; Left: Raw points in a single study cell over DTM, local origin of the cell is marked with the yellow dot; Middle & Right: VRML models of individual tree crowns in the study cell visualized from different view directions, individual tree crowns are marked with different colours.

Each detected tree crown is described by an array of 2D tree crown regions in different layers at the different height levels. Since the layers in voxel space have certain thickness, 3D prisms can be constructed for the 2D crown regions in different layers with the thickness of layers as the height of the prisms. A prismatic 3D crown model can be reconstructed by a combination of all the crown prisms at the different height levels (Figure 12. (a)).

For an individual crown, the following parameters are available:

● Height of the tree;
● Height range of the crown;
● Diameters of the crown at the different height levels;
● The largest diameter of the tree crown and its correspondent height;
● Volume of the crown.

**Figure 13.**
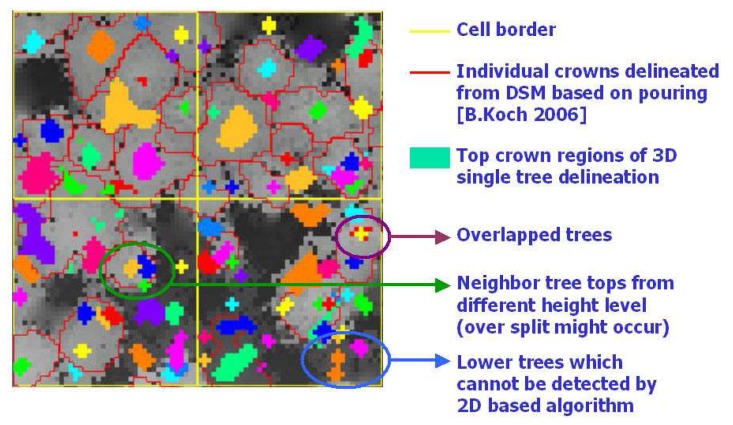
Comparison between 3D single tree modelling and DSM based single tree delineation.

Figure 12. (b) shows the final result of 3D single tree modelling in a study cell. To evaluate the result, a comprehensive reference data set based on field measurements is still missing. A superficial evaluation can be accomplished by a comparison with DSM and the results from DSM based single tree delineation method (Figure 13). It is quite obvious that the 3D single tree modelling is more capable of detecting lower trees at the first canopy layer that are relatively difficult for the 2D DSM based algorithm. Trees from lower canopy layer overlapped by the higher canopies are also detected, which is impossible for the DSM based algorithms. The coarse comparison also shows the risk of over split with big tree crowns raised by the 3D single tree modelling.

## 4. Conclusion

The statistical method seems to be efficient and reliable in detecting the existence and height range of canopy layers due to the first visual checks. Further detailed verifications respected to the field inventory results will be delivered in the next stage of our study.

For the 3D single tree modelling, the advantage of our algorithm is that not only the individual trees whose crowns are at the top canopy layer, but also the lower trees and even trees at sub canopy layers whose crowns are covered by the top canopy layer are extracted. The crown contours delineated from the different height levels of an individual tree crown provides a high approximation between the 3D crown model and the reality. More meaningful parameters such as crown height range and volume are achieved from the crown model.

Considering the obvious difference between shapes of tree top regions, it is strongly indicated that conifers will have a small top region because of their cone-shaped crowns while deciduous will present a large top region due to their sphere-shaped crowns. The potential of a classification of tree species based on the 3D crown models is noticeable.

The main problem of current algorithm is that for big trees with more than one crown peak, the crown might be over split. The parameters such as the scales of the voxel space and the size of morphological elements are highly influential to the results. Detailed studies on those parameters are necessary. Another disadvantage is that the segmentation of study cells might split the trees along the border of the cells, which can be improved by an enlargement of study cell size or a substitution of the raster grid by a moving window for study cell segmentation.

The main factors which influence the quality of the 3D single tree modelling are the density of the raw point clouds and the situation of the forest stand. Higher point density will improve the accuracy of tree extraction. Better result can be expected for a lower canopy closure forest stand.

Further studies will be concentrated on the detailed verifications of the algorithm and utilizations of 3D crown models in forest inventory.

## References

[1] Andersen H.E., Foster J.R, Reutebuch S.E. (2003). Estimating forest structure parameters on Fort Lewis Military Reservation using airborne laser scanner (LIDAR) data.

[2] Harding D., Lefsky M., Parker G., Blair J. (2001). Laser altimeter canopy height profiles: Methods and validation for closed-canopy, broadleaf forests. Remote Sensing of The Environment.

[3] Holmgren J., Jonsson T., Thies M., Koch B., Spiecker H., Weinacker H. (2004). Large scale airborne laser-scanning of forest resources in Sweden. Remote Sensing and Spatial Information Sciences.

[4] Hyyppä J., Inkinen M. (1999). Detecting and estimating attributes for single trees using laser scanner. The Photogrammetric Journal of Finland.

[5] Koch B., Heyder U., Weinacker H. (2006). Detection of individual tree crowns in airborne lidar data. Photogrammetric Engineering and Remote Sensing.

[6] Lefsky M., Cohen W., Acker S., Parker G., Spies T., Harding D. (1999). Lidar remote sensing of biophysical properties and canopy structure of forests of Douglas-fir and western hemlock. Remote Sensing of Environment.

[7] Lim K., Treitz P., Baldwin K., Morrison I., Green J. (2003). Lidar remote sensing of biophysical properties of tolerant northern hardwood forests. Canadian Journal of Remote Sensing.

[8] Næsset E. (2002). Predicting forest stand characteristics with airborne scanning laser using a practical two-stage procedure and field data. Remote Sensing of Environment.

[9] Persson Å., Holmgren J., Söderman U. (2002). Detecting and measuring individual trees using an airborne laser scanner. Photogrametric Engineering and Remote Sensing.

[10] Pyysalo U., Hyyppä H. (2002). Reconstructing tree crowns from laser scanner data for feature extraction. Commission III Symposion Graz.

[11] Reitberger J., Krzystek P., Stilla U. (2007). Combined tree segmentation and stem detection using full waveform Lidar data. Laser Scanning and SilviLaser.

[12] Weinacker H., Koch B., Weinacker R. (2004). TREESVIS- A software system for simultaneous 3D-Real-Time visualization of DTM, DSM, Laser row data, Multi-spectral data, simple tree and building models. Remote Sensing and Spatial Information Sciences.

